# Human Septal Cartilage Tissue Engineering: Current Methodologies and Future Directions

**DOI:** 10.3390/bioengineering11111123

**Published:** 2024-11-07

**Authors:** Tammy B. Pham, Robert L. Sah, Koichi Masuda, Deborah Watson

**Affiliations:** 1 Department of Otolaryngology-Head and Neck Surgery, UC San Diego Health, La Jolla, CA 92093, USA; tbpham@health.ucsd.edu; 2 Shu Chien-Gene Lay Department of Bioengineering, UC San Diego Jacobs School of Engineering, La Jolla, CA 92093, USA; rsah@ucsd.edu; 3 Department of Orthopedic Surgery, UC San Diego Health, La Jolla, CA 92093, USA; komasuda@health.ucsd.edu

**Keywords:** nasal septal cartilage, cartilage tissue engineering, cartilage scaffolds, bioink, 3D printed cartilage

## Abstract

Nasal septal cartilage tissue engineering is a promising and dynamic field with the potential to provide surgical options for patients with complex reconstruction needs and mitigate the risks incurred by other tissue sources. Developments in cell source selection, cell expansion, scaffold creation, and three-dimensional (3D) bioprinting have advanced the field in recent years. The usage of medicinal signaling cells and nasal chondroprogenitor cells can enhance chondrocyte proliferation, stimulate chondrocyte growth, and limit chondrocyte dedifferentiate. New scaffolds combined with recent innovations in 3D bioprinting have allowed for the creation of more durable and customizable constructs. Future developments may increase technical accessibility and manufacturability, and lower costs, to help incorporate these methods into pre-clinical studies and clinical applications of septal cartilage tissue engineering.

## 1. Introduction

Nasal septal cartilage is a critical support structure in the human nasal framework [1,2]. Nasal framework deformities and nasal obstruction due to septal cartilage collapse or loss can result from trauma, tumors, aging, or previous surgery. Typical current options for reconstruction of the nasal framework include autologous, allogenic, or synthetic sources [3]. While autologous nasal septal cartilage is widely used in head and neck reconstructive surgery and is a preferred tissue source for the straightforward surgical patient, its use is often limited by its finite availability, difficulties in shaping and maintaining graft structure over time, and donor site morbidity [4]. More complex surgical patients include those who have large nasal defects involving multiple cartilage structures (e.g., due to tumor resection), those undergoing revision surgery and thus lack sufficient autologous septal cartilage for harvest, or some combination of both factors. Other autologous sources include auricular cartilage and costal cartilage, which carry inherent risks of donor site morbidity and long-term complications including warping or resorption, which may necessitate revision surgery [5]. Allogenic sources bear the risks of immune rejection, disease transmission, and resorption, while synthetic or combination synthetic-autologous sources also have the potential to provoke immune response, infection, or extrusion [3,4]. Nasal septal cartilage tissue engineering is a promising and dynamic field with the potential to provide surgical options for patients with complex reconstruction needs and mitigate these other risks incurred with existing tissue sources [6,7].

While the fundamentals of tissue engineering were initially developed several decades ago, novel developments in cell source selection, cell expansion, scaffold creation, and three-dimensional (3D) bioprinting have served to advance the field in recent years. This review serves to incorporate the literature in PubMed and Web of Science for articles on “septal cartilage tissue engineering”, “3D printing hydrogel”, and “3D printing cartilage” as well as articles either citing or cited by these articles that were deemed relevant to this topic. Our review synthesizes relevant work particularly related to nasal septal cartilage and published within the last decade. Other notable recent reviews include those by Vertu-Ciolino et al. [8] on the current challenges in nasal cartilage tissue engineering, including biocompatibility requirements and degradability of implants, by Cao et al. [9] highlighting advances in 3D bioprinting techniques, and by Huang et al. [10] on the role of hydrogels in 3D bioprinting. Our review expands on these existing resources to provide a conceptual understanding of the current strategies employed in tissue engineering for nasal cartilage reconstruction and recent advancements in translational efforts that can bring septal cartilage tissue engineering into the clinical setting with attention to in vivo trials and 3D bioprinting.

## 2. Composition, Structure, and Function of Human Nasal Septal Cartilage

**Figure 1:**
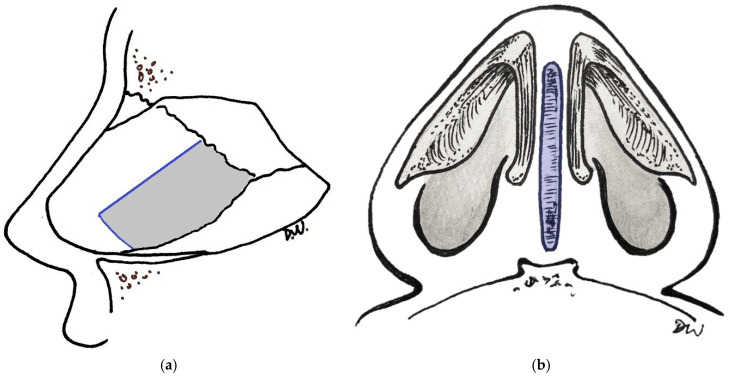
Nasal cartilage schematics: (**a**) L-strut preservation during septal cartilage harvest (gray represents harvested area); (**b**) columellar strut graft (in blue) placed in a precise pocket between the two medial crura of the lower lateral cartilages.

Septal cartilage serves as a major support for the nasal framework [2]. Cartilage is a relatively acellular tissue comprising chondrocytes, which are embedded within and together maintain the surrounding hydrated extracellular matrix (ECM). The ECM includes matrix macromolecules such as collagen and proteoglycan, and it serves as a depot by binding regulatory factors such as insulin-like growth factor 1 (IGF-1) and transforming growth factor β (TGF-β) [11]. Septal cartilage is a quadrilateral piece of hyaline cartilage that separates the two nasal cavities and articulates with upper and lower lateral cartilages dorsally and caudally, the perpendicular plate of the ethmoid bone posterosuperiorly, and with the vomer bone posteroinferiorly [1]. The major collagen in septal cartilage is type II collagen, although there are also type I collagen and elastin fibers in septal perichondrium [12,13]. Compositional analyses of cadaveric human nasal septal cartilage indicate that each milligram of wet cartilage contains 24,900 ± 3910 cells, 73.9 ± 6.4 micrograms of collagen, 17.1 ± 3.0 micrograms of sulfated glycosaminoglycan (sGAG) [14], and 77.7 ± 1.9% water [15]. Chondrocytes, which are responsible for synthesizing and maintaining cartilage, account for only ~1% of the volume of mature adult tissue [16]. When examining the spatial variation in composition of human nasal septal cartilage, Neuman et al. found that components of collagen and sGAG are distributed non-uniformly across the cartilaginous septum, with higher collagen content in caudal than cephalad septal segments and higher sGAG levels in ventral than dorsal segments [14]. Advanced age has been associated with a reduction in sGAG and cell content at rates of 7.7% and 7.4%, respectively, per decade of life [15]. This highlights the significance of preserving at least a 1 cm L-strut (Figure 1a) during rhinoplasty and other nasal reconstructive procedures where septal cartilage is harvested in order to preserve the collagen-rich caudal septum and to maintain nasal framework support, especially as patients age [14].

Human nasal septal cartilage has mechanical material properties that contribute to its function as a structural support. Its mechanical properties are anisotropic, with direction-varying properties such as compressive modulus that is higher in the vertical and caudal-cephalic orientations than in the medial-lateral orientation; hydraulic permeability tends also to be greatest in the latter orientation [17]. This has relevance in the orientation of septal cartilage grafts when used in critical support areas. One such example is the columellar strut graft (Figure 1b), which is placed in a precise pocket between the two medial crura of the lower lateral cartilages (adjacent to the caudal-most edge of the septum) and serves to support the nasal tip and maintain tip projection [18]. Biomechanical testing to assess the tensile Young modulus (*E*) of nasal septum, another index of the material’s stiffness, shows substantial variability between patients. In one study, *E* was found to be 4.8, 39.3, and 32.8 MPa in three patients, although the authors stated that the first patient’s cartilage needed to be trimmed for measurement and this may have resulted in the lower value [19]. In a study comparing the bending properties of native and tissue-engineered septal cartilage, three-point bending tests showed linear load-displacement curves with a greater stiffness (0.19 ± 0.15 N/mm vs. 0.014 ± 0.019 N/mm) and bending modulus (1.97 ± 1.25 MPa vs. 0.32 ± 0.25 MPa) for the native septal cartilage compared to the tissue-engineered constructs, respectively [20]. These biomechanical material properties of nasal septal cartilage contribute to its function as a nasal framework support and serve as benchmarks targeted by efforts in cartilage tissue engineering.

## 3. Current Methodologies and Advancements in Cell Source Selection, Chondrocyte Expansion, Chondrocyte Redifferentiation, and Scaffold Strategies

Refinements to each step of the existing cartilage tissue engineering methodologies have allowed for steady progress toward the goal of achieving surgically implantable tissue-engineered cartilage constructs [6]. The most common and current in vitro human septal cartilage tissue engineering paradigm entails (1) an initial harvest of autologous chondrogenic cells from the patient, (2) tissue processing to isolate the chondrocytes from the ECM, (3) expansion of the chondrocyte cells in a monolayer culture—during which the cells dedifferentiate to a fibroblastic phenotype [21], (4) redifferentiation of cells in a three-dimensional culture, (5) in vitro incubation within a scaffold, and, finally, (6) transfer back to the patient [6,22].

### 3.1. Cell Source Selection

A major step in cartilage tissue engineering is selecting a cell source. Common sources are autologous chondrocytes from the nasal septum, auricle, or rib. Naumann et al. [23] described an in vitro macroaggregate culture system technique using nasoseptal and auricular cartilage and found that engineered nasoseptal cartilage demonstrated GAG content higher than auricular cartilage, though still less than native cartilage. In an analysis of cell yield, proliferation, and post-expansion differentiation, Tay et al. [24] found that cell yield was highest from auricular cartilage, followed by nasal and rib cartilage, respectively. However, in the presence of platelet-derived growth factor bb (PDGF-BB), nasal chondrocytes displayed superior post-expansion chondrogenic potential compared to ear cartilage. Overall, as a cell source for septal cartilage tissue engineering, nasal cartilage and auricular cartilage appear to be preferable compared to rib cartilage [24].

Although chondrocytes seem to be the ideal candidate cells for cartilage tissue engineering, such cells have disadvantages. These include the need to harvest autologous cartilage from the patient in advance of reconstruction (which can be logistically taxing and can induce scarring and deformity). In addition, chondrocytes tend to lose their phenotypic characteristics and show fibroblast-like features after just three monolayer culture passages [25].

Alternatives to autologous chondrocytes include pluripotent stem cells, medicinal signaling cells (MSCs; previously named mesenchymal stem cells) [26], and more differentiated chondroprogenitor cells [22].

Pluripotent stem cells include both embryonic stem cells (ESCs) and induced pluripotent stem cells (iPSCs). ESCs are advantageous because of their unlimited self-renewal and potential to differentiate into almost any cell type; chondrogenic differentiation of ESCs has had some success [27]; however, there remains significant ethical debate over their usage. iPSCs result from reprograming somatic cells with specific transcription factors to generate pluripotent stem cells that have shown some chondrogenic capacity [28]. However, there remain concerns regarding tumorigenicity and safety of iPSCs [29].

MSCs can be derived from a variety of sources, including bone marrow or adipose tissue. The advantages of MSCs include rapid growth and longevity. However, disadvantages include senescence with repeated sub-cultivation and the tendency for MSC-induced chondrogenesis to produce type I collagen-rich fibrocartilage tissue, rather than hyaline cartilage, which leads to inferior long-term functional outcomes [22,30]. Some studies have investigated using mixtures of MSCs with chondrocytes to enhance chondrocyte proliferation [31,32].

Investigations into the use of nasal chondroprogenitor cells (CPCs), which were first isolated from human nasal septal cartilage in 2011 [33], have uncovered opportunities for in situ nasal septal cartilage regeneration. CPCs express pluripotency and mesoectodermal stem cell markers and can proliferate rapidly in vitro with higher clonogenic potential and longer lifespan compared to other studied cells [33,34]. In a recent review of CPCs, only nine studies thus far have isolated human nasal CPCs and investigated their features and functions against other cell types. Furthermore, Vinod et al. [35] investigated CPCs isolated using different protocols and found that migratory CPCs demonstrated superior chondrogenic potential and higher levels of GAG deposition compared to fibronectin adhesion assay-derived CPCs. Further development of migratory CPCs may allow for more hyaline-like repair tissues for reconstruction.

### 3.2. Chondrocyte Expansion

When using chondrocytes that have been isolated from autologous or allogenic donor cartilage tissue, a key step in cartilage tissue engineering typically involves chondrocyte expansion. This is most commonly carried out using a monolayer culture to increase the cell yield; however, the process often leads to chondrocyte dedifferentiation and an increase in type I collagen relative to type II collagen, especially after multiple monolayer culture passes [36]. These alterations in matrix composition may limit the mechanical stability of neocartilage constructs [21]. In response, researchers have sought different ways to reduce dedifferentiation during chondrocyte expansion, including selection of specific culture media. Fetal bovine serum (FBS) is a common media supplement in cartilage tissue engineering. However, human serum generally provides equivalent or more potent stimulation of chondrocyte proliferation and subsequent chondrogenesis [37,38]. Furthermore, De Angelis et al. [39] found that incorporating platelet lysate in cultured articular chondrocytes resulted in a reduction in dedifferentiation during expansion compared to fetal bovine serum (FBS), and Kachroo et al. [40] found that platelet lysate was also helpful as a growth supplement for the expansion of articular chondroprogenitors.

### 3.3. Chondrocyte Redifferentiation

Many strategies to modulate chondrocyte redifferentiation have been studied, including the use of growth factors, oxygen tension, and scaffolds for 3D cultures [41].

**Growth Factors**: Numerous growth factors have been studied as part of a strategy to promote chondrocyte redifferentiation. Various combinations of IGF-1, fibroblast growth factor (FGF), the TGF-β family, and the bone morphogenetic protein (BMP) family have been shown to increase proliferation, enhance matrix deposition, and result in higher levels of GAG and type II collagen accumulation in expanded human nasal septal chondrocytes [42,43,44]. Similarly, a growth factor cocktail of TGF-β1, bone morphogenetic proteins 2 and 6 (BMP2 and BMP6), growth differentiation factor-5 (GDF5), and FGF2 were used to promote osteochondral differentiation of expanded human periosteum-derived stem/progenitor cells [45].

**Oxygen Tension**: The oxygen concentration in human septal cartilage is reduced relative to that in the inferior turbinate and ambient oxygen levels [46]. Twu et al. [47] showed that expansion of human septal chondrocytes in monolayer culture and GAG accumulation were greatest at normoxic oxygen tension but that both normoxic and hypoxic cultures of human septal chondrocytes embedded in alginate beads supported robust ECM deposition. Research in articular chondrocytes in physiologically hypoxic (“physoxic”) conditions showed improved expression of chondrocytic markers and suppression of dedifferentiation markers [48].

**Hyaluronidase Treatment**: Watson et al. [49] found that hyaluronidase treatment of engineered septal cartilage decreased total sGAG content without inhibiting expansive growth of the constructs. Decreased sGAG in treated constructs resulted in increased collagen-to-sGAG ratios and was associated with an increase in tensile strength and stiffness.

### 3.4. Scaffold-Based and Scaffold-Free Strategies

The goal of utilizing scaffolds in scaffold-based techniques is to provide a three-dimensional framework to support cell attachment and proliferation and to mirror the mechanical properties of native cartilage. The ideal scaffolds are (1) biocompatible with the proliferating cells, (2) minimally immunogenic, (3) easily shaped to fit the reconstructive needs of the patient, and (4) mechanically stable over time [50]. Table 1 summarizes the properties of commonly used scaffold materials, their advantages and disadvantages, and advances in the development of each to help offset some of those issues.

Both natural and synthetic materials have been utilized as scaffolds for septal cartilage tissue engineering. Natural scaffolds such as collagen are biocompatible with low immunogenicity. Furthermore, they tend to support proliferation and prevent dedifferentiation. Mao et al. [51] utilized decellularized chondrocyte ECM and found that chondrocytes cultured on this medium proliferated faster and dedifferentiated less. However, one drawback of natural scaffolds is that their degradation over time can be rapid. On the other hand, synthetic scaffolds such as Polycaprolactone (PCL), Poly L-lactic acid (PLLA), Polyglycolic Acid (PGA), and Poly(lactic-co-glycolic acid) (PLGA) can be easily produced in a customized manner with variable degradation times [52]. However, they, or their breakdown products, can cause foreign body reactions, worsen dedifferentiation, and reduce cell proliferation. As a result, several techniques have utilized composite combinations of both natural and synthetic scaffold material (“hybrid scaffolds”) to balance the advantages and disadvantages of each [53]. Other composite techniques, such as adding fibrin glue to synthetic scaffolds, have been described and shown to increase cell proliferation and maintain ECM production [54,55].

The disadvantages to scaffold-based approaches include the potential for immune rejection of the scaffold material and scaffold material degradation [56]. In contrast, scaffold-free approaches do not rely on solid supports, use only cells to sustain the developing tissue ECM [56,57], and aim to mimic native cartilage development patterns [58]. Several scaffold-free culture techniques have been described [58,59,60,61]. Chia et al. [59] used the scaffold-free alginate-recovered-chondrocyte (ARC) method to produce tissue-engineered cartilage. In this method, cells were initially cultured in alginate and subsequently released from the alginate, seeded onto a semipermeable membrane system, and integrated in a three-dimensional configuration. These ARC constructs showed superior structural stability and histologic and gross appearance of cartilaginous tissue compared to monolayer constructs. Dobratz et al. [60] performed scaffold-free in vivo culture of human nasal septal chondrocytes suspended in alginate. After up to 38 weeks of culture, the retrieved explants were noted to have similar histology and type II collagen content as native septal cartilage. More recent studies have utilized scaffold-free approaches with ESCs [61] and other cell sources [58] for the development of tissue-engineered articular cartilage as well. Future developments of these approaches have the potential to advance the field of tissue engineering by circumventing many of the limitations we currently face with the classic scaffold-based approaches.

**Table 1:** Scaffolds for cartilage tissue engineering [7,22,55].

Scaffold	Properties	Pros	Cons	Opportunities for Improvement
**Natural Scaffolds**				
Collagen	Natural matrix polymer	Biocompatible Low immunogenicity Facilitates cell adhesion and proliferation	Rapid degradation	Increasing cross-linked collagen scaffolds [62] Composite combinations with synthetic scaffolds to reduce degradation rate [63]
Alginate	Natural polysaccharide extracted from sea algae	Easily crosslinked Compatible with 3D bioprinting	Poor cellular infiltration and attachment	Addition of fibronectin and matrigel coating improed cell attachment to honeycomb alginate scaffolds [64] Addition of collagen increases cell proliferation and ECM production [65,66]
Hyaluronic Aid	Anionic polysaccharide	Facilitates cell proliferation	Poor mechanical strength even when crosslinked	Composite combinations with synthetic scaffolds to improve mechanical strength [67]
Decellularized ECM	Comprises proteoglycans and collagen	Biocompatible	Difficult for cells to reseed due to ECM density	Decreasing ECM density via pulverization or creation of porous channels [68,69]
**Synthetic Scaffolds**				
Polycaprolactone (PCL)	Low melting point Hydrophobic	High mechanical stability Low melting point Excellent blend-compatibility with different additives Hydrophobic with longer degradation time	Suboptimal cell attachment and tissue integration	Composite combinations with natural scaffolds to improve biocompatibility [70]
Poly-L-lactic Acid (PLLA)	Biodegradable thermoplastic polyester	High mechanical stability	Suboptimal cell attachment and tissue integration	Composite combinations with natural scaffolds to improve biocompatibility [71]
Polyglycolic Acid (PGA)	Biodegradable thermoplastic polyester	High mechanical stability	Suboptimal cell attachment and tissue integration	Composite combinations with natural scaffolds to improve biocompatibility [72] Addition of fibrin glue to chondrocytes seeded onto a PGA scaffold results in increased cellular proliferation while maintaining production of ECM components [55]
Poly(lactic-co-glycolic acid) (PLGA)	Biodegradable polyester	High mechanical stability Excellent blend-compatibility with different additives	Suboptimal cell attachment and tissue integration	Composite combinations with natural scaffolds to improve biocompatibility [63]

## 4. Current Methods in Three-Dimensional Bioprinting

Three-dimensional (3D) printing employs 3D computer models and additive manufacturing to create structures using a variety of substrates [73]. Bioprinting for cartilage tissue engineering typically employs living cells and extracellular matrix raw materials as a “bioink” to produce living tissues under sterile conditions [9]. While autologous cartilage grafts are often sufficient for simple nasal reconstruction cases, these grafts are limited in both quantity and donor site geometry. Successful implementation of 3D bioprinting may allow for the creation of tissues that can be designed to fit a complex surgical patient’s specific defect and reconstructive needs, which can be invaluable in cases pertaining to patients with large composite nasal defects from tumor resection [74]. Table 2 outlines the general schema for 3D printing for cartilage tissue engineering.

The first step of 3D printing for tissue reconstruction is to define the target and create a 3D computer model. Mapping of the defect can be based on a combination of physical exam and measurement of the defect as well as advanced imaging techniques such as computerized tomography (CT) or magnetic resonance imaging (MRI) [9]. One must then generate a 3D computer-aided design (CAD) model of the reconstruction needed to repair that defect and restore form and function to the nose.

The second step is to select a bioink material. The ideal bioink is biocompatible, conducive to printability, and has desirable mechanical properties. There exist several reviews [76,77] of natural and synthetic bioinks that have been used in cartilage tissue engineering. As is the case with traditional scaffold material selection, natural polymers tend to have low cytotoxicity, high biocompatibility, and a resemblance to the native ECM but suffer from batch-to-batch variations and inferior mechanical properties; in contrast, synthetic materials have superior mechanical properties but higher cytotoxicity and worse biocompatibility [78]. Natural polymers can be formed into hydrogels, 3D polymer matrices that can expand through water absorption, promoting cell proliferation, and providing structural support in a way that can replicate the functions of native ECM [79]. Alginate are commonly used natural polymers [80] that can form hydrogels in an ionic cross-linking approach; however, despite their non-immunogenicity, non-toxicity, and good printability [76,81], they tend to have poor mechanical properties and need to be combined with another polymer such as collagen, chitosan, agarose, or gelatin [76]. In particular, while chitosan is a natural polymer similar to GAGs in the ECM and is otherwise an excellent scaffold material from a structural integrity perspective, it is limited in its ability to allow for chondrocyte attachment and proliferation [75]. Recent work [82] using tissue-specific decellularized ECM for articular cartilage lesion repair suggests the potential for incorporating decellularized ECM into bioinks for 3D printed scaffolds which may better promote chondrogenesis. Other recent formulations of bioinks such as those using blends of carboxylated agarose and native agarose have successfully yielded bioinks with improved high printability, high biocompatibility, high stiffness, and the ability to generate complex structures with high cell densities [83].

The third step is to select a 3D printing technique (e.g., extrusion bioprinting, inkjet, laser-assisted bioprinting, stereolithography) [78]. Extrusion-based bioprinting is the most widely utilized technique in cartilage tissue engineering due to its ease of use. However, it induces substantial mechanical stress on the bioink during the extrusion process, which can reduce cell viability and tissue functionality. Inkjet printing is restricted to only materials with low viscosity. It has the advantage of being able to deliver even small volumes of liquid in a controlled manner. In cartilage tissue engineering, it can be utilized to deposit cells onto the scaffold. Laser-assisted bioprinting is a more advanced method that does not induce mechanical stress on the tissues and can achieve high levels of resolution [84]. However, its use is limited by high cost and technical complexity. Related stereolithography methods with micromirror devices also deliver light patterns and activate photocurable polymers. Aisenbrey et al. [85] used stereolithography-based 3D printing of a hybrid scaffold filled with a cell-laden hydrogel that was used to repair a focal articular chondral defect. While the use of stereolithography is promising and a relatively recent development, its use is currently limited by a lack of suitable hydrogels with which it could be used [78].

Finally, the fourth step of bioprinting is to select a cell source and incorporate it into the bioprinted scaffold [9,78]. A 2021 systematic review [86] of sixteen papers on 3D bioprinting of scaffolding for nasal cartilage defects showed a variety of techniques and cellular sources. Of the eleven translational research studies, ten were in animal models and one was in humans [87]. In terms of cell selection, most studies used harvested chondrocytes (from human nasal septal/alar cartilage, goat/porcine auricular cartilage, and rabbit knee articular cartilage) while one study [88] used nasal chondroprogenitor cells. The most common 3D printing method utilized was the fused filament fabrication method (FFFM) [86]. The FFFM uses a thermoplastic filament and is easy to use with a variety of available biomaterials. However, a disadvantage is the high manufacturing temperature, which can limit the use of some cells [89].

## 5. Pre-Clinical In Vivo Studies and Clinical Applications of Tissue-Engineered Septal Cartilage

In general, pre-clinical research has shown that neocartilage constructs are generally well tolerated. In a pilot study of the murine model, Chang et al. [90] investigated the in vivo biocompatibility of septal neocartilage constructs developed in vitro by an alginate intermediate step. The eight mice in their study were found to tolerate neocartilage construct implantation well, without evidence of infection or extrusion, and the histologic, biochemical, and biomechanical features of implanted constructs closely resembled native septal tissue when compared with preimplant constructs [90].

In a landmark study, Fulco et al. [91] published a first-in-human trial of five patients who underwent the excision of alar lobule non-melanoma skin cancer and subsequently received engineered cartilage grafts which were then implanted with accompanying paramedian forehead or nasolabial flap. For these patients, 6 mm cartilage biopsy samples from the nasal septum were obtained under local anesthesia at the time of initial tumor biopsy. The chondrocytes were isolated from the cartilage biopsies, expanded, seeded, and cultured with autologous serum onto Chondro-Gide (a porcine-derived type I/III collagen membrane) over the course of four weeks, resulting in 25 × 25 × 2 mm grafts. At six months after implantation, during a flap refinement surgery as the last stage, a 3 × 3 mm biopsy of the implanted graft was obtained for analysis. These biopsy samples of reconstructed tissues histologically displayed fibromuscular fatty structures typical of the alar lobule. At 12 months post-implantation, all patients reported satisfaction with their aesthetic and functional breathing outcomes (found to be no different than pre-operatively), pain scores were 0 for all patients, and there were no reported adverse events. The cutaneous sensation and structural stability of the reconstructed nostrils were clinically satisfactory and found to not significantly differ from those of the opposite unoperated nostril, and the overall airflow resistance was only slightly higher compared to the opposite unoperated nostril. Overall, this study demonstrated a feasible and functionally successful approach to alar reconstruction using tissue engineered from harvested nasal septal cartilage with promising implications for similar applications for larger defects in the future.

While the in-human trial by Fulco et al. [91] using human nasal chondrocytes on the Chondro-Gide collagen scaffold was a very promising demonstration of the use of tissue-engineered septal cartilage, these commercially available scaffolds come in a limited number of standard sizes, and while they could in theory be constructed into various 3D shapes, there remains the challenge of maintaining the appropriate mechanical properties and design to allow integration into the body. The successful development of a technique to 3D print tissue-engineered cartilage would be especially promising for patients with complex or large nasal defects.

There is precedence for the use of 3D printing in nasal reconstructive surgery. Byrne and Garcia [74] described the use of a 3D-printed intraoperative surgical guide for the reconstruction of complex, subtotal, or total nasal defects. The patients in their study had large nasal defects due to surgical resection of a neoplasm or due to trauma. These patients were referred to an anaplastologist who created a mold of the patient’s nose and then designed a reconstructed model using wax. This model was then approved by the patient and scanned into a 3D computer model and 3D printed into a custom-made translucent surgical guide which was then sterilized for intraoperative use. This intraoperative guide was used to help reconstruct the nose using an autologous rib and ear cartilage and a combination of flaps for the cutaneous reconstruction. Further developments in 3D printing of tissue-engineered cartilage may allow for further advances in the reconstruction of large nasal defects such as these, with the potential for 3D printed cartilage to replace rib cartilage, which would reduce donor site morbidity, forgo the challenges of carving rib cartilage into the perfect custom shape for reconstruction, and, as a result, possibly decrease operative time and complexity.

## 6. Pre-Clinical In Vivo Studies of 3D Printed Tissue-Engineered Septal Cartilage

Advancements in 3D printing have allowed for the improved customization of implant geometry to match the anatomic needs of the patient. Some techniques utilize synthetic 3D printed scaffolds seeded with chondrocytes. In a murine model, Xu et al. [92] used 3D printing to create and implant a PGA/PLLA scaffold seeded with chondrocytes which on analysis at eight weeks post-implantation were found to mirror the shape and biomechanical properties of the native lower lateral cartilages.

Other studies have explored the use of natural scaffolds to provide a familiar environment for chondrocytes and enhance chondrocyte attachment. In a study comprising 24 rabbits, Shokri et al. [93] utilized 3D printed elastin–gelatin–hyaluronic acid scaffolds with chondrocytes for in vivo regeneration of nasal septal cartilage defects with successful reduction of residual defect mean area. Apelgren et al. [31] similarly used nanofibrillated cellulose and alginate bioink and an extrusion 3D bioprinter to create a scaffold; however, this time, they investigated using human nasal chondrocytes (hNCs) alone versus with the addition of human bone marrow-derived MSCs (hBM-MSCs). They compared cell mixes of either hNCs alone, hBM-MSCs alone, a mixture of 20% hNCs and 80% hBM-MSCs, and an acellular wash. The scaffolds were then washed in one of the four cell mediums and subsequently implanted into mice. After 30–60 days, an analysis of the sectioned explanted constructs showed progressive chondrogenesis and proliferation of GAG-positive cells in both the hNC and mixed groups, while almost none were found in the hBM-MSC group and none were found in the acellular group. Furthermore, data showed that the mixed group had a higher proliferative capacity compared to the hNC-only group despite starting with only 20% of the original concentration of the hNC-only group, suggesting that the addition of hBM-MSCs enhanced chondrocyte proliferation.

Advancements in CAD software have allowed for the creation of high-fidelity 3D models from 2D image input. Using this technique, Yi et al. [94] generated 3D facial models from front and side view 2D facial images, which were then transformed into custom nasal implant models. They used projection-based microstereolithography to create 3D-printed custom nasal implant scaffolds using PCL, which were then injected with a mixture of human adipose-derived stem cells and either an alginate hydrogel or cartilage-derived hydrogel. These nasal cartilage constructs were then subcutaneously implanted into 4-week-old mice. An evaluation of these tissues at 6 and 12 weeks post-implantation showed that the cartilage-derived hydrogel group showed faster formation of cartilage tissues and higher proteoglycan and GAG production compared to the alginate hydrogel group.

To take 3D bioprinting one step further, Lan et al. [95] utilized the Freeform Reversible Embedding of Suspended Hydrogel (FRESH) bioprinting technique [96,97] to create customizable and functional cartilage matrix-engineered nasal cartilage using chondrocyte-laden bioink. While type I collagen is very biocompatible and used extensively in cartilage tissue engineering, it is typically a poor bioink due to its low viscosity, low elastic modulus, and slow gelation time. FRESH allows for the 3D printing of materials with low elastic modulus such as alginate, collagen, and fibrin by utilizing a secondary hydrogel that serves as a temporary, thermoreversible, and biocompatible support. Lan et al. [95] used the FRESH technique with a bioink comprising with hNC-laden bovine type I collagen hydrogel and showed that this mixture exhibited favorable printability characteristics. In their in vitro analysis after six weeks, they found that the 3D printed nasal cartilage constructs displayed biochemical, and histological characteristics akin to native nasoseptal cartilage, with GAG levels in the constructs closely resembling that of native cartilage and evidence of chondrocytes that formed round lacuna structures (indicating successful redifferentiation of hNCs into chondrocyte phenotype) [95]. However, this study did not test the mechanical properties of the cartilage constructs compared to native cartilage.

Subsequently, Lan et al. [98] mapped patient-specific lower lateral cartilages from CT scans and utilized the 3D printed nasal cartilage constructs in vivo in mice to assess the long-term biochemical and mechanical properties of those explanted constructs. Their study showed noninferiority to tissue-engineered human nasal cartilage grafts created using the Chondro-Gide scaffold. Notably, the chondrogenic capacity of the hNCs was higher when suspended in the hydrogel compared to the Chondro-Gide porcine-derived type I/III collagen membrane. Furthermore, the technique utilized allows for customizable and patient-specific, 3D-printed engineered cartilage constructs.

## 7. Conclusions

Septal cartilage tissue engineering remains a promising area of research with the ability to revolutionize precision medicine for complex surgical patients (for example, those patients undergoing revision nasal surgery or surgery to repair large nasal defects) for whom the standard reconstruction techniques fall short. There remain several areas where further advancement could help bring ongoing research into the clinical realm. The first is an adequate autologous cell source. The greater and faster the expansion of cells to the appropriate type, the fewer would be needed from the harvested cartilage at the time of surgery. Specialized media and bioreactors can help achieve this. In addition, while cartilage progenitor cells may provide a more plentiful cell source, controlling their growth is also necessary. The second area is establishing effective scaffolds and bioinks. Ideally, scaffolds will interact appropriately with the chosen cell type, in terms of cell adhesion, proliferation, and redifferentiation. In addition, the scaffold would provide appropriate mechanical properties for reconstruction. Hybrid scaffolds appear particularly promising. Finally, further advancements in translational models are needed to test the effectiveness of new designs for septal cartilage tissue engineering.

## Figures and Tables

**Table 2:** Three-dimensional (3D) printing for cartilage tissue engineering [9,10,75].

Steps/Decision Points		
1	Modeling the Defect and Custom Graft	Physical Exam Measurement of Defect CT/MRI Creation of CAD Model
2	Selection of Bioink for Scaffold	Natural (e.g., natural polymers, hydrogels) Synthetic Composite
3	Selection of 3D Printing Technique	Extrusion-based Inkjet Laser-Assisted Stereolithography
4	Selection of Cell Source	Chondrocytes Chondroprogenitor Cells Stem Cells Co-Cultures of Cell Sources (e.g., chondrocytes + stem cells)

3D: Three-dimensional; CT: Computed tomography; MRI: Magnetic resonance imaging; CAD: Computer-aided design.

## Abbreviations

3D	three-dimensional
ARC	alginate-recovered-chondrocyte
CAD	computer-aided design
CT	computed tomography
ECM	extracellular matrix
FFFM	fused filament fabrication method
FRESH	freeform reversible embedding of suspended hydrogel
GAG	glycosaminoglycan
hBM	human bone marrow
hNC	human nasal chondrocytes
IGF-1	insulin-like growth factor 1
MRI	magnetic resonance imaging
MSC	medicinal signaling cell
PCL	Polycaprolactone
PGA	Polyglycolic acid
PLGA	Poly(lactic-co-glycolic acid)
PLLA	Poly L-lactic acid
TGF-β	transforming growth factor β
